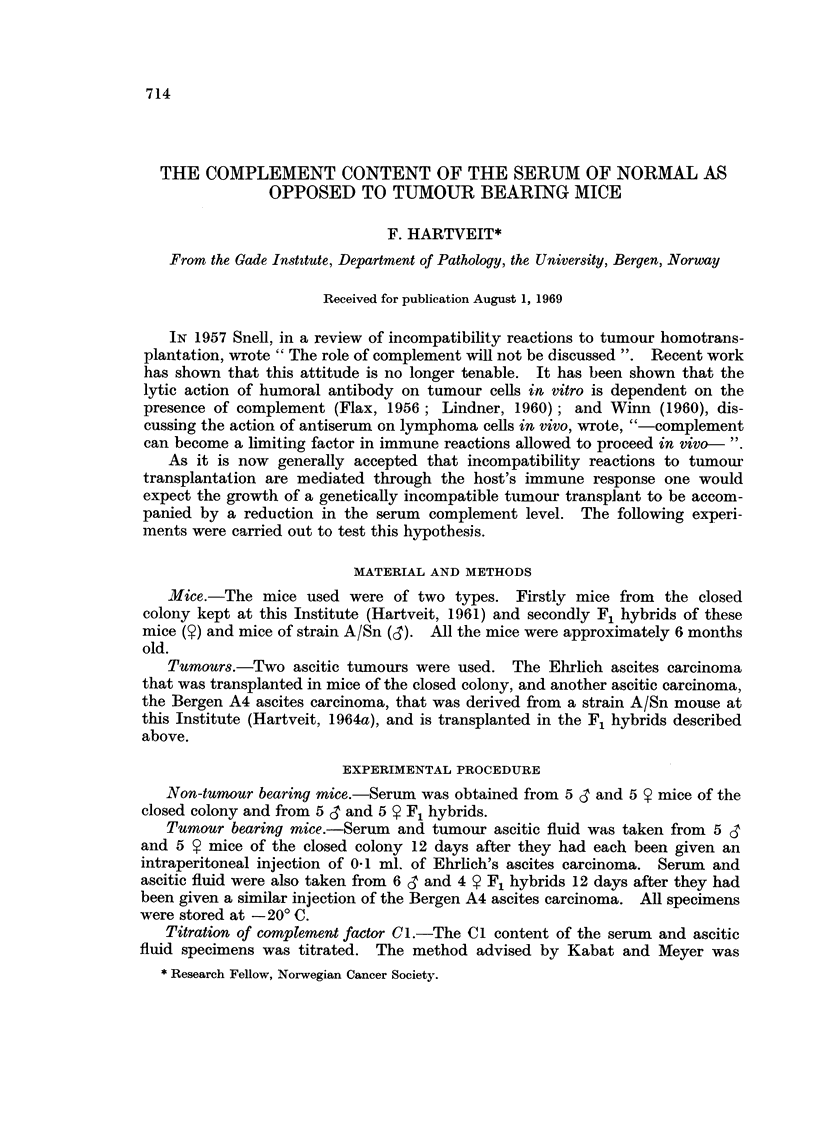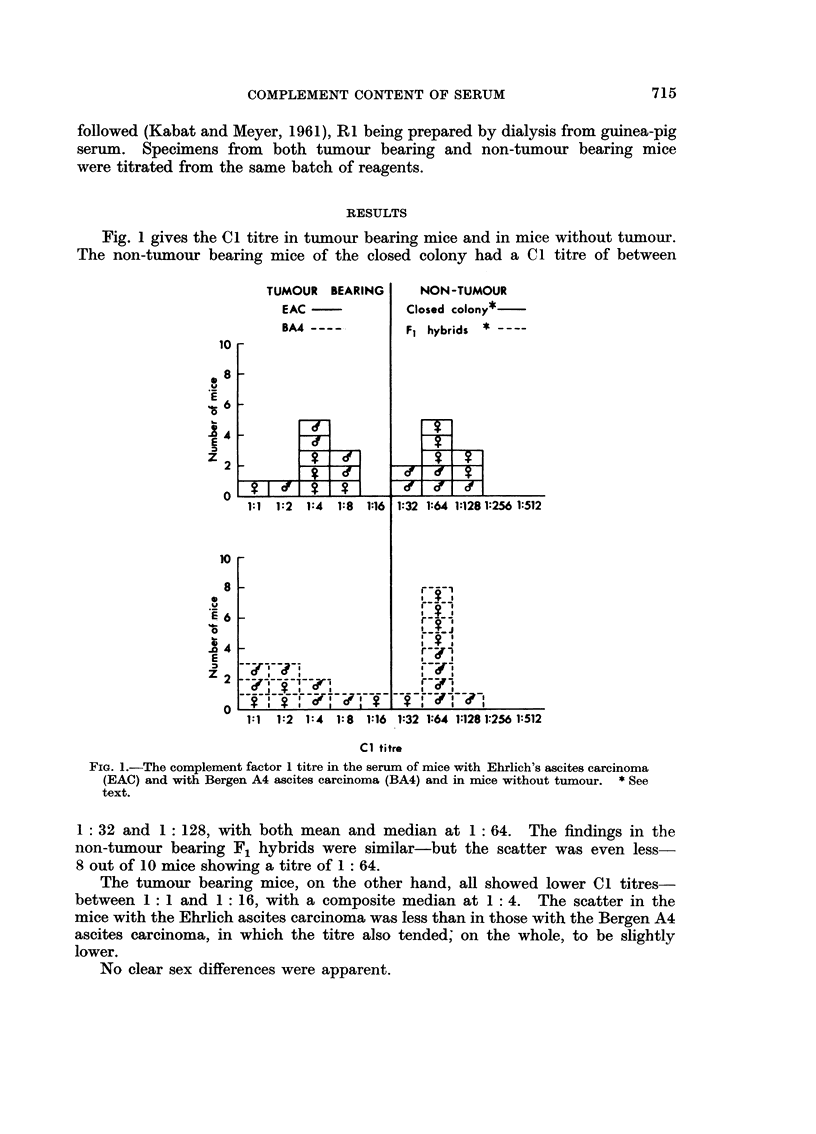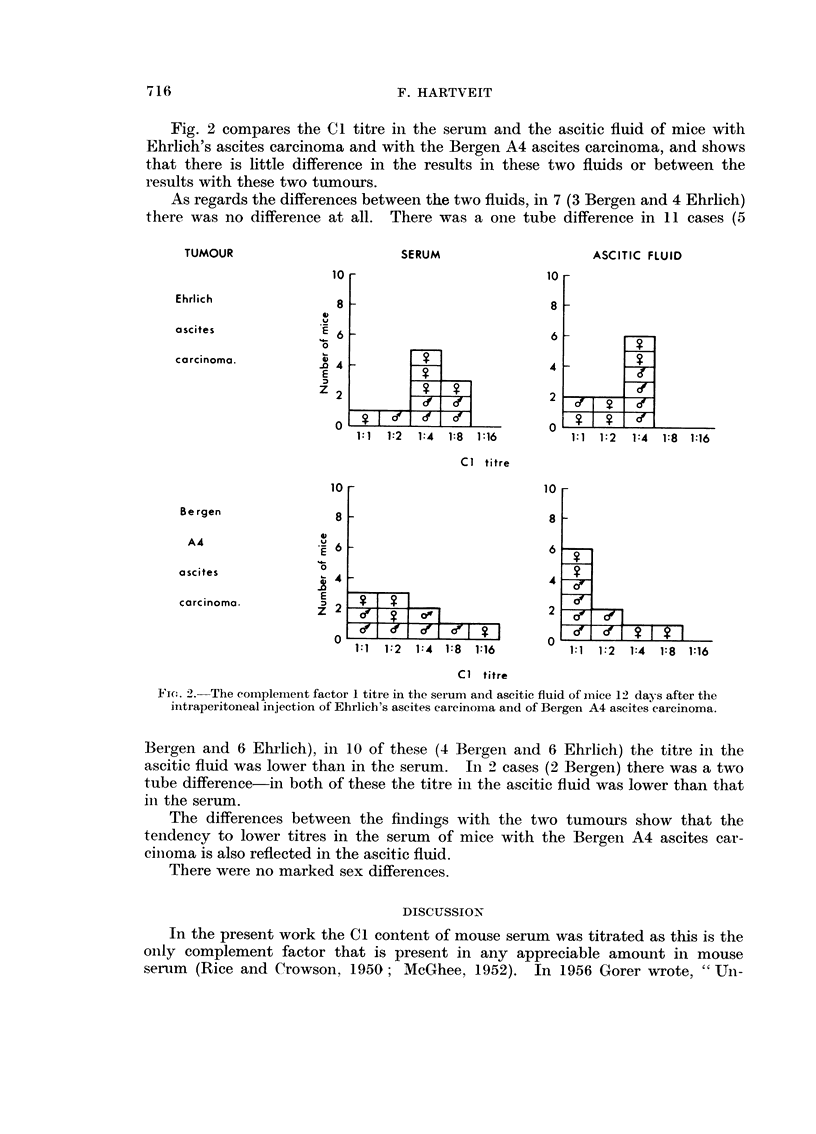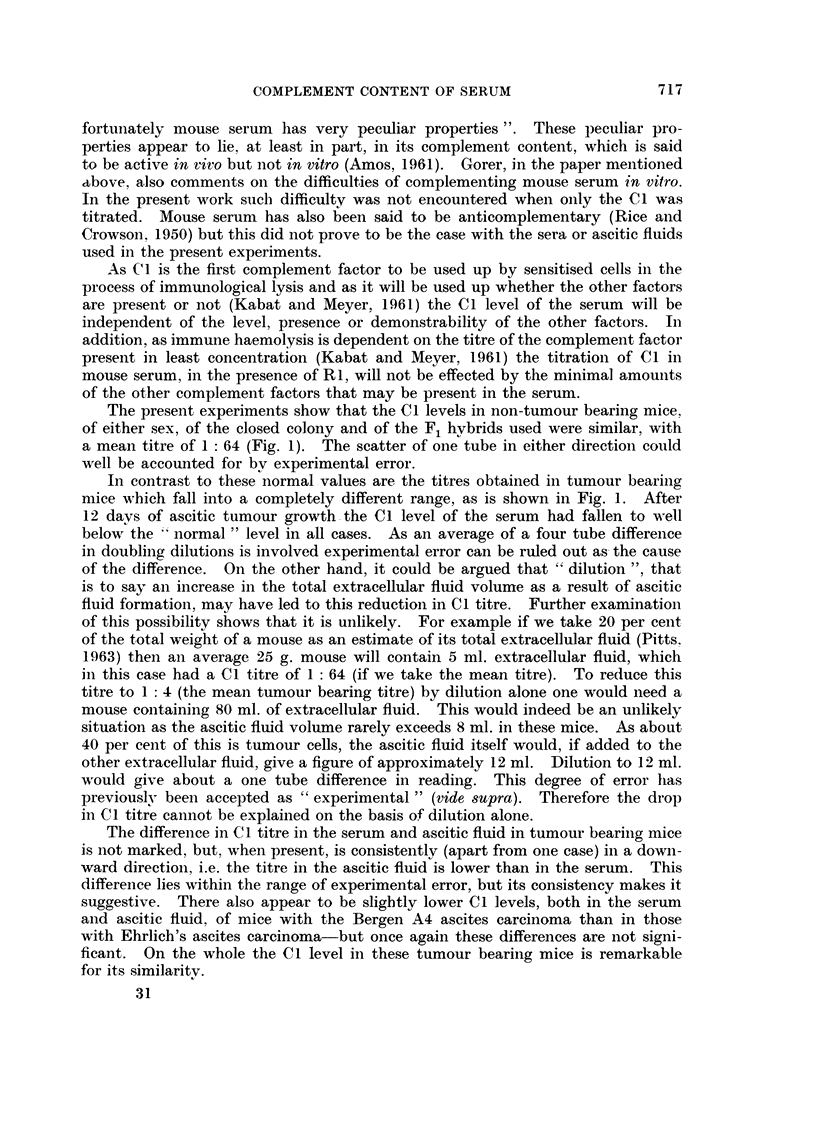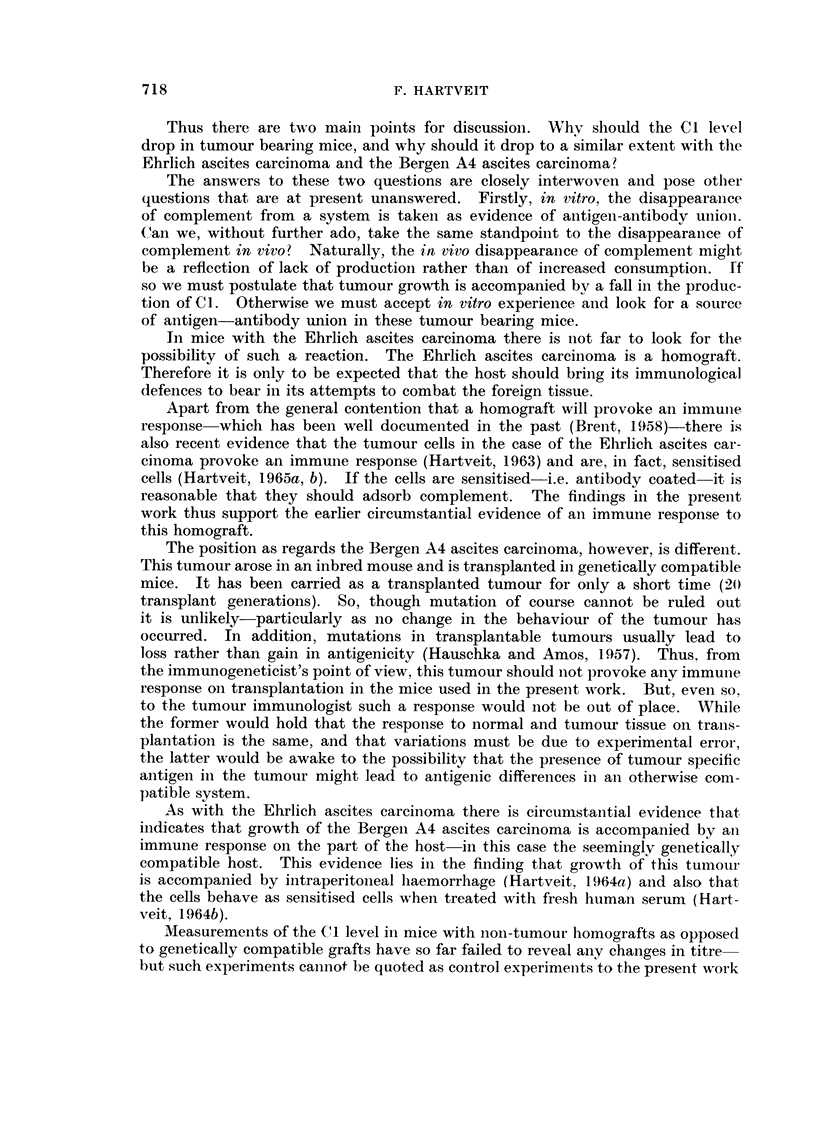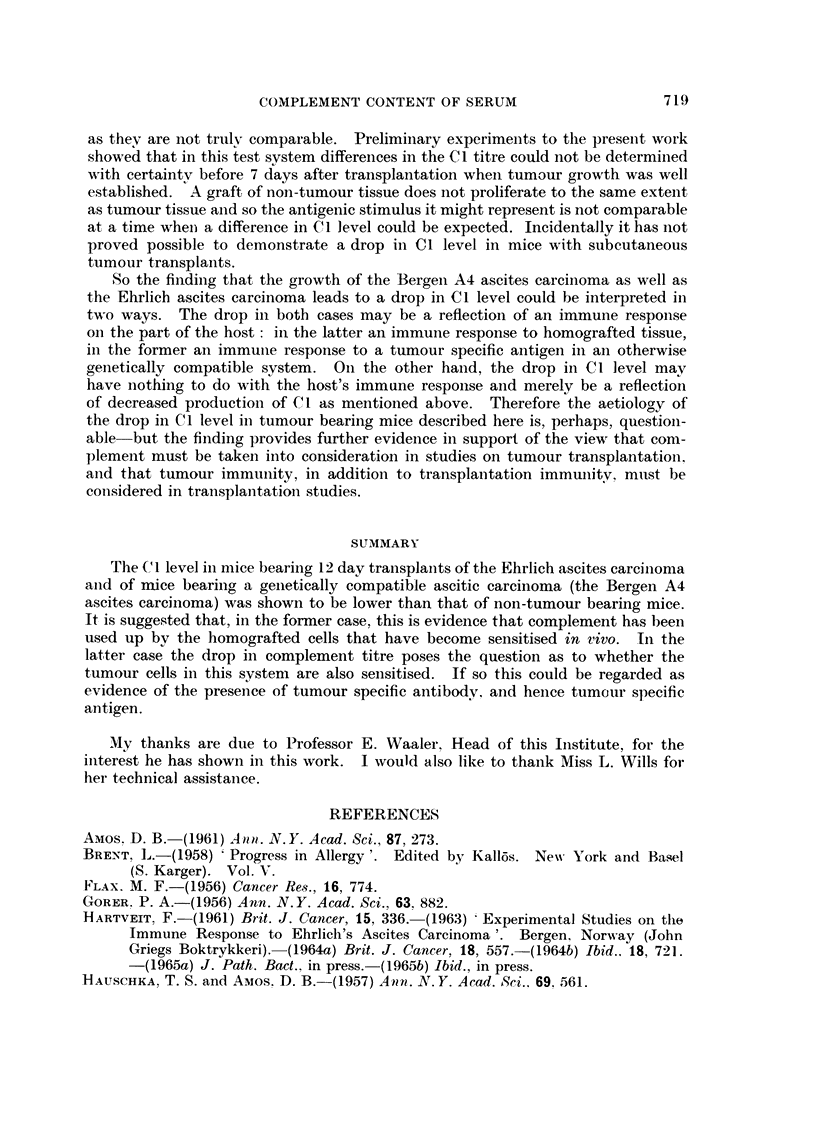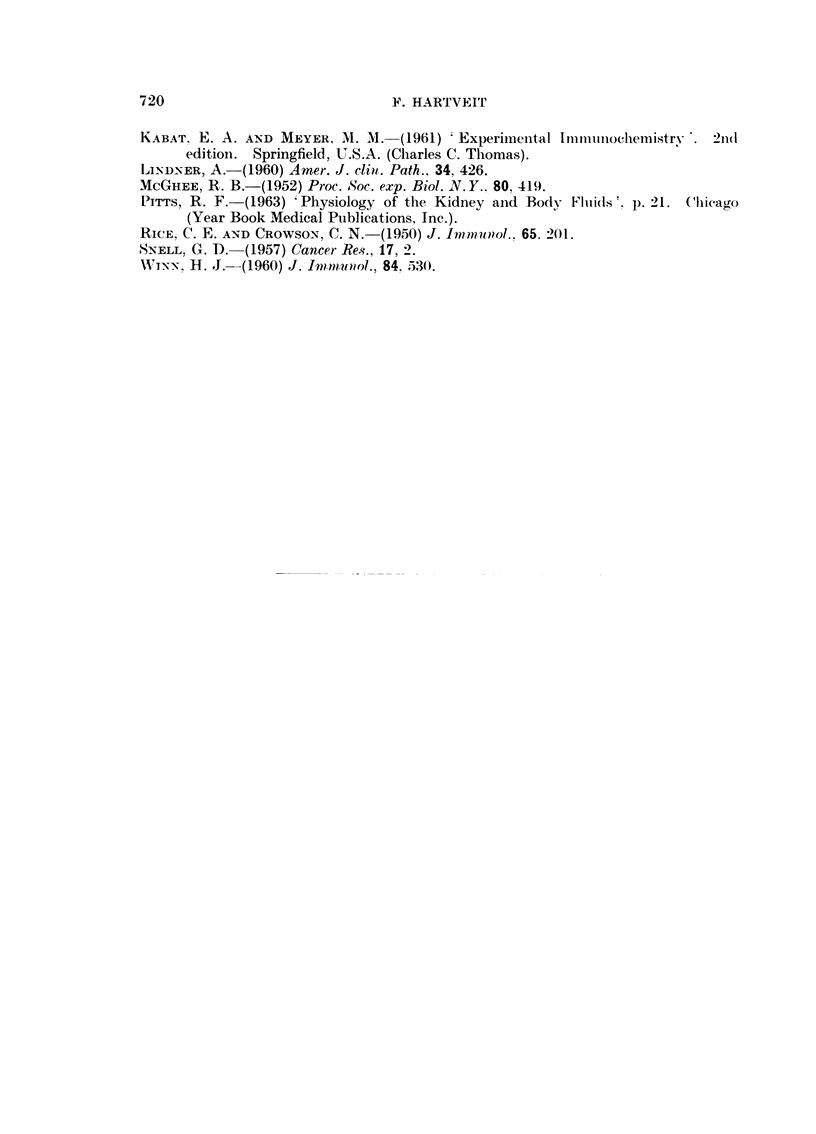# The Complement Content of the Serum of Normal as Opposed to Tumour Bearing Mice

**DOI:** 10.1038/bjc.1964.82

**Published:** 1964-12

**Authors:** F. Hartveit


### THE COMPLEMENT CONTENT OF THE SERUM OF NORMAL AS OPPOSED TO TUMOUR BEARING MICE

F. HARTVEIT*

*From the Gade Institute, Department of Pathology, the University, Bergen, Norway*

Received for publication August 1, 1969

IN 1957 Snell, in a review of incompatibility reactions to tumour homotransplantation, wrote " The role of complement will not be discussed ". Recent work has shown that this attitude is no longer tenable. It has been shown that the lytic action of humoral antibody on tumour cells *in vitro* is dependent on the presence of complement (Flax, 1956 ; Lindner, 1960) ; and Winn (1960), discussing the action of antiserum on lymphoma cells *in vivo*, wrote, "—complement can become a limiting factor in immune reactions allowed to proceed *in vivo*— ".

As it is now generally accepted that incompatibility reactions to tumour transplantation are mediated through the host's immune response one would expect the growth of a genetically incompatible tumour transplant to be accompanied by a reduction in the serum complement level. The following experiments were carried out to test this hypothesis.

### MATERIAL AND METHODS

*Mice.*—The mice used were of two types. Firstly mice from the closed colony kept at this Institute (Hartveit, 1961) and secondly $F_1$ hybrids of these mice (♀) and mice of strain A/Sn (♂). All the mice were approximately 6 months old.

*Tumours.*—Two ascitic tumours were used. The Ehrlich ascites carcinoma that was transplanted in mice of the closed colony, and another ascitic carcinoma, the Bergen A4 ascites carcinoma, that was derived from a strain A/Sn mouse at this Institute (Hartveit, 1964*a*), and is transplanted in the $F_1$ hybrids described above.

### EXPERIMENTAL PROCEDURE

*Non-tumour bearing mice.*—Serum was obtained from 5 ♂ and 5 ♀ mice of the closed colony and from 5 ♂ and 5 ♀ $F_1$ hybrids.

*Tumour bearing mice.*—Serum and tumour ascitic fluid was taken from 5 ♂ and 5 ♀ mice of the closed colony 12 days after they had each been given an intraperitoneal injection of 0·1 ml. of Ehrlich's ascites carcinoma. Serum and ascitic fluid were also taken from 6 ♂ and 4 ♀ $F_1$ hybrids 12 days after they had been given a similar injection of the Bergen A4 ascites carcinoma. All specimens were stored at $-20°$ C.

*Titration of complement factor C*1.—The C1 content of the serum and ascitic fluid specimens was titrated. The method advised by Kabat and Meyer was

* Research Fellow, Norwegian Cancer Society.

followed (Kabat and Meyer, 1961), R1 being prepared by dialysis from guinea-pig serum. Specimens from both tumour bearing and non-tumour bearing mice were titrated from the same batch of reagents.

### RESULTS

Fig. 1 gives the C1 titre in tumour bearing mice and in mice without tumour. The non-tumour bearing mice of the closed colony had a C1 titre of between

FIG. 1.—The complement factor 1 titre in the serum of mice with Ehrlich's ascites carcinoma (EAC) and with Bergen A4 ascites carcinoma (BA4) and in mice without tumour. * See text.

1 : 32 and 1 : 128, with both mean and median at 1 : 64. The findings in the non-tumour bearing $F_1$ hybrids were similar—but the scatter was even less—8 out of 10 mice showing a titre of 1 : 64.

The tumour bearing mice, on the other hand, all showed lower C1 titres—between 1 : 1 and 1 : 16, with a composite median at 1 : 4. The scatter in the mice with the Ehrlich ascites carcinoma was less than in those with the Bergen A4 ascites carcinoma, in which the titre also tended; on the whole, to be slightly lower.

No clear sex differences were apparent.

Fig. 2 compares the C1 titre in the serum and the ascitic fluid of mice with Ehrlich's ascites carcinoma and with the Bergen A4 ascites carcinoma, and shows that there is little difference in the results in these two fluids or between the results with these two tumours.

As regards the differences between the two fluids, in 7 (3 Bergen and 4 Ehrlich) there was no difference at all. There was a one tube difference in 11 cases (5

FIG. 2.—The complement factor 1 titre in the serum and ascitic fluid of mice 12 days after the intraperitoneal injection of Ehrlich's ascites carcinoma and of Bergen A4 ascites carcinoma.

Bergen and 6 Ehrlich), in 10 of these (4 Bergen and 6 Ehrlich) the titre in the ascitic fluid was lower than in the serum. In 2 cases (2 Bergen) there was a two tube difference—in both of these the titre in the ascitic fluid was lower than that in the serum.

The differences between the findings with the two tumours show that the tendency to lower titres in the serum of mice with the Bergen A4 ascites carcinoma is also reflected in the ascitic fluid.

There were no marked sex differences.

### DISCUSSION

In the present work the C1 content of mouse serum was titrated as this is the only complement factor that is present in any appreciable amount in mouse serum (Rice and Crowson, 1950; McGhee, 1952). In 1956 Gorer wrote, "Unfortunately mouse serum has very peculiar properties ". These peculiar properties appear to lie, at least in part, in its complement content, which is said to be active *in vivo* but not *in vitro* (Amos, 1961). Gorer, in the paper mentioned above, also comments on the difficulties of complementing mouse serum *in vitro*. In the present work such difficulty was not encountered when only the C1 was titrated. Mouse serum has also been said to be anticomplementary (Rice and Crowson, 1950) but this did not prove to be the case with the sera or ascitic fluids used in the present experiments.

As C1 is the first complement factor to be used up by sensitised cells in the process of immunological lysis and as it will be used up whether the other factors are present or not (Kabat and Meyer, 1961) the C1 level of the serum will be independent of the level, presence or demonstrability of the other factors. In addition, as immune haemolysis is dependent on the titre of the complement factor present in least concentration (Kabat and Meyer, 1961) the titration of C1 in mouse serum, in the presence of R1, will not be effected by the minimal amounts of the other complement factors that may be present in the serum.

The present experiments show that the C1 levels in non-tumour bearing mice, of either sex, of the closed colony and of the $F_1$ hybrids used were similar, with a mean titre of 1 : 64 (Fig. 1). The scatter of one tube in either direction could well be accounted for by experimental error.

In contrast to these normal values are the titres obtained in tumour bearing mice which fall into a completely different range, as is shown in Fig. 1. After 12 days of ascitic tumour growth the C1 level of the serum had fallen to well below the " normal " level in all cases. As an average of a four tube difference in doubling dilutions is involved experimental error can be ruled out as the cause of the difference. On the other hand, it could be argued that " dilution ", that is to say an increase in the total extracellular fluid volume as a result of ascitic fluid formation, may have led to this reduction in C1 titre. Further examination of this possibility shows that it is unlikely. For example if we take 20 per cent of the total weight of a mouse as an estimate of its total extracellular fluid (Pitts, 1963) then an average 25 g. mouse will contain 5 ml. extracellular fluid, which in this case had a C1 titre of 1 : 64 (if we take the mean titre). To reduce this titre to 1 : 4 (the mean tumour bearing titre) by dilution alone one would need a mouse containing 80 ml. of extracellular fluid. This would indeed be an unlikely situation as the ascitic fluid volume rarely exceeds 8 ml. in these mice. As about 40 per cent of this is tumour cells, the ascitic fluid itself would, if added to the other extracellular fluid, give a figure of approximately 12 ml. Dilution to 12 ml. would give about a one tube difference in reading. This degree of error has previously been accepted as " experimental " (*vide supra*). Therefore the drop in C1 titre cannot be explained on the basis of dilution alone.

The difference in C1 titre in the serum and ascitic fluid in tumour bearing mice is not marked, but, when present, is consistently (apart from one case) in a downward direction, i.e. the titre in the ascitic fluid is lower than in the serum. This difference lies within the range of experimental error, but its consistency makes it suggestive. There also appear to be slightly lower C1 levels, both in the serum and ascitic fluid, of mice with the Bergen A4 ascites carcinoma than in those with Ehrlich's ascites carcinoma—but once again these differences are not significant. On the whole the C1 level in these tumour bearing mice is remarkable for its similarity.

Thus there are two main points for discussion. Why should the C1 level drop in tumour bearing mice, and why should it drop to a similar extent with the Ehrlich ascites carcinoma and the Bergen A4 ascites carcinoma?

The answers to these two questions are closely interwoven and pose other questions that are at present unanswered. Firstly, *in vitro*, the disappearance of complement from a system is taken as evidence of antigen-antibody union. Can we, without further ado, take the same standpoint to the disappearance of complement *in vivo*? Naturally, the *in vivo* disappearance of complement might be a reflection of lack of production rather than of increased consumption. If so we must postulate that tumour growth is accompanied by a fall in the production of C1. Otherwise we must accept *in vitro* experience and look for a source of antigen—antibody union in these tumour bearing mice.

In mice with the Ehrlich ascites carcinoma there is not far to look for the possibility of such a reaction. The Ehrlich ascites carcinoma is a homograft. Therefore it is only to be expected that the host should bring its immunological defences to bear in its attempts to combat the foreign tissue.

Apart from the general contention that a homograft will provoke an immune response—which has been well documented in the past (Brent, 1958)—there is also recent evidence that the tumour cells in the case of the Ehrlich ascites carcinoma provoke an immune response (Hartveit, 1963) and are, in fact, sensitised cells (Hartveit, 1965*a*, *b*). If the cells are sensitised—i.e. antibody coated—it is reasonable that they should adsorb complement. The findings in the present work thus support the earlier circumstantial evidence of an immune response to this homograft.

The position as regards the Bergen A4 ascites carcinoma, however, is different. This tumour arose in an inbred mouse and is transplanted in genetically compatible mice. It has been carried as a transplanted tumour for only a short time (20 transplant generations). So, though mutation of course cannot be ruled out it is unlikely—particularly as no change in the behaviour of the tumour has occurred. In addition, mutations in transplantable tumours usually lead to loss rather than gain in antigenicity (Hauschka and Amos, 1957). Thus, from the immunogeneticist's point of view, this tumour should not provoke any immune response on transplantation in the mice used in the present work. But, even so, to the tumour immunologist such a response would not be out of place. While the former would hold that the response to normal and tumour tissue on transplantation is the same, and that variations must be due to experimental error, the latter would be awake to the possibility that the presence of tumour specific antigen in the tumour might lead to antigenic differences in an otherwise compatible system.

As with the Ehrlich ascites carcinoma there is circumstantial evidence that indicates that growth of the Bergen A4 ascites carcinoma is accompanied by an immune response on the part of the host—in this case the seemingly genetically compatible host. This evidence lies in the finding that growth of this tumour is accompanied by intraperitoneal haemorrhage (Hartveit, 1964*a*) and also that the cells behave as sensitised cells when treated with fresh human serum (Hartveit, 1964*b*).

Measurements of the C1 level in mice with non-tumour homografts as opposed to genetically compatible grafts have so far failed to reveal any changes in titre—but such experiments cannot be quoted as control experiments to the present work as they are not truly comparable. Preliminary experiments to the present work showed that in this test system differences in the C1 titre could not be determined with certainty before 7 days after transplantation when tumour growth was well established. A graft of non-tumour tissue does not proliferate to the same extent as tumour tissue and so the antigenic stimulus it might represent is not comparable at a time when a difference in C1 level could be expected. Incidentally it has not proved possible to demonstrate a drop in C1 level in mice with subcutaneous tumour transplants.

So the finding that the growth of the Bergen A4 ascites carcinoma as well as the Ehrlich ascites carcinoma leads to a drop in C1 level could be interpreted in two ways. The drop in both cases may be a reflection of an immune response on the part of the host : in the latter an immune response to homografted tissue, in the former an immune response to a tumour specific antigen in an otherwise genetically compatible system. On the other hand, the drop in C1 level may have nothing to do with the host's immune response and merely be a reflection of decreased production of C1 as mentioned above. Therefore the aetiology of the drop in C1 level in tumour bearing mice described here is, perhaps, questionable—but the finding provides further evidence in support of the view that complement must be taken into consideration in studies on tumour transplantation, and that tumour immunity, in addition to transplantation immunity, must be considered in transplantation studies.

### SUMMARY

The C1 level in mice bearing 12 day transplants of the Ehrlich ascites carcinoma and of mice bearing a genetically compatible ascitic carcinoma (the Bergen A4 ascites carcinoma) was shown to be lower than that of non-tumour bearing mice. It is suggested that, in the former case, this is evidence that complement has been used up by the homografted cells that have become sensitised *in vivo*. In the latter case the drop in complement titre poses the question as to whether the tumour cells in this system are also sensitised. If so this could be regarded as evidence of the presence of tumour specific antibody, and hence tumour specific antigen.

My thanks are due to Professor E. Waaler, Head of this Institute, for the interest he has shown in this work. I would also like to thank Miss L. Wills for her technical assistance.

### REFERENCES

Amos, D. B.—(1961) *Ann. N.Y. Acad. Sci.*, **87**, 273.

Brent, L.—(1958) 'Progress in Allergy'. Edited by Kallõs. New York and Basel (S. Karger). Vol. V.

Flax, M. F.—(1956) *Cancer Res.*, **16**, 774.

Gorer, P. A.—(1956) *Ann. N.Y. Acad. Sci.*, **63**, 882.

Hartveit, F.—(1961) *Brit. J. Cancer*, **15**, 336.—(1963) 'Experimental Studies on the Immune Response to Ehrlich's Ascites Carcinoma'. Bergen, Norway (John Griegs Boktrykkeri).—(1964*a*) *Brit. J. Cancer*, **18**, 557.—(1964*b*) *Ibid.*, **18**, 721.—(1965*a*) *J. Path. Bact.*, in press.—(1965*b*) *Ibid.*, in press.

Hauschka, T. S. and Amos, D. B.—(1957) *Ann. N.Y. Acad. Sci.*, **69**, 561.

KABAT, E. A. AND MEYER, M. M.—(1961) 'Experimental Immunochemistry'. 2nd edition. Springfield, U.S.A. (Charles C. Thomas).

LINDNER, A.—(1960) *Amer. J. clin. Path.*, **34**, 426.

MCGHEE, R. B.—(1952) *Proc. Soc. exp. Biol. N.Y.*, **80**, 419.

PITTS, R. F.—(1963) 'Physiology of the Kidney and Body Fluids', p. 21. Chicago (Year Book Medical Publications, Inc.).

RICE, C. E. AND CROWSON, C. N.—(1950) *J. Immunol.*, **65**, 201.

SNELL, G. D.—(1957) *Cancer Res.*, **17**, 2.